# Analysis of archived residual newborn screening blood spots after whole genome amplification

**DOI:** 10.1186/s12864-015-1747-2

**Published:** 2015-08-13

**Authors:** Brandi L. Cantarel, Yunping Lei, Daniel Weaver, Huiping Zhu, Andrew Farrell, Graeme Benstead-Hume, Justin Reese, Richard H. Finnell

**Affiliations:** Baylor Health, Baylor Institute for Immunology Research, 3434 Live Oak Street, Dallas, TX 75204 USA; Department of Nutritional Sciences, Dell Pediatric Research Institute, The University of Texas at Austin, 1400 Barbara Jordan Blvd, Austin, TX 78723 USA; Genformatic, 6301 Highland Hills Drive, Austin, TX 78731 USA; Present address: Asuragen Inc, 2150 Woodward Street #100, Austin, TX 78744 USA; Department of Biology, Boston College, Boston, MA 02467 USA

## Abstract

**Background:**

Deidentified newborn screening bloodspot samples (NBS) represent a valuable potential resource for genomic research if impediments to whole exome sequencing of NBS deoxyribonucleic acid (DNA), including the small amount of genomic DNA in NBS material, can be overcome. For instance, genomic analysis of NBS could be used to define allele frequencies of disease-associated variants in local populations, or to conduct prospective or retrospective studies relating genomic variation to disease emergence in pediatric populations over time. In this study, we compared the recovery of variant calls from exome sequences of amplified NBS genomic DNA to variant calls from exome sequencing of non-amplified NBS DNA from the same individuals.

**Results:**

Using a standard alignment-based Genome Analysis Toolkit (GATK), we find 62,000–76,000 additional variants in amplified samples. After application of a unique kmer enumeration and variant detection method (RUFUS), only 38,000–47,000 additional variants are observed in amplified gDNA. This result suggests that roughly half of the amplification-introduced variants identified using GATK may be the result of mapping errors and read misalignment.

**Conclusions:**

Our results show that it is possible to obtain informative, high-quality data from exome analysis of whole genome amplified NBS with the important caveat that different data generation and analysis methods can affect variant detection accuracy, and the concordance of variant calls in whole-genome amplified and non-amplified exomes.

**Electronic supplementary material:**

The online version of this article (doi:10.1186/s12864-015-1747-2) contains supplementary material, which is available to authorized users.

## Background

Since the release of the DNA sequence of the first human genome in 2000, which ushered in the shift from Sanger sequencing to next-generation sequencing (NGS), thousands of human genomes and exomes have now been sequenced. The promise of human exome and genome analysis is to generate sufficient information to enable clinicians to provide patients with personalized medical care. Yet, each human exome can contain as many as a million single nucleotide variants (SNVs) when compared to the human reference genome. In order to determine which variants are most clinically relevant for an individual patient, it may be necessary to determine the frequency of minor alleles - not only in the general population, but in the specific population to which each patient belongs. Thus, deep sequencing of large numbers samples in highly differentiated and admixed populations is critical. The 1000 Genomes Project (1000G) aims to provide genome sequences for over 3000 individuals from several distinct populations from across the globe [1]. The UK10K [2], is another genome sequencing project with an objective to sequence over 10,000 human genomes from different populations across the United Kingdom. Similarly, the NHLBI Exome Sequencing Project (ESP) [3] has generated exome sequencing data from thousands of individuals with the goal of identifying genes and variants that contribute to heart, lung and blood disorders. The ESP and UK10K cohorts contain individuals with disease and aim to define the relationship between phenotype and genotype, and associate genomic variation with disease risk, therapeutic safety, and efficacy and patient outcomes.

Yet despite great effort to sequence thousands of individuals, current data alone may not be sufficient to allow clinicians to distinguish which variants are informative in local patient populations. The 1000G Project has chosen to sequence greater numbers of individuals at very low sequencing depth—but with the potential risk that the data contains more sequencing errors and therefore less accurate allele frequency information. Meanwhile, the UK10G will generate greater numbers of sequences at greater sequencing depth, but the represented populations are limited to those more prevalent in the UK. Similarly, the individuals in the ESP are more limited in their populations of origin, and are skewed towards individuals with a heart, lung or blood disorder. For the physician interested in personalized medicine, a more accurate metric would be a variant’s frequency in the local population of a state, county or city—the population in Lancaster County, PA, with large Amish contributions, might have an allele frequency spectrum substantially different than the local population in Travis County, TX, where significant Mexican-American and African-American populations are present.

One resource available for allele frequency determinations in the local population is banked newborn bloodspot samples routinely collected and used for Mendelian disease screening of neonates using metabolite profiling. These de-identified blood samples could serve as a source for the determination of variant frequencies in a local area. Yet, these samples are less than ideal because of their age and limited amount of genomic DNA they contain. Previous work has shown that these samples can be indeed sequenced using whole exome sequencing [4]. Here we assess the effects of whole genome amplification on our ability to identify real variation in exome sequence data as an application for samples with limited material. Specifically, we are interested in the number of single nucleotide variants (SNVs) that are attributed to the amplification process compared to technical duplication. Additionally, we have also examined the differences in variant sets identified from the same sample when using two different commercially available exome capture kits. Our data should encourage more extensive utilization of NBS specimen archived around the globe for a variety of clinical and research applications.

## Methods

The study was approved by the institutional review boards of University of Texas at Austin (study#: 2010-10-0110) and Texas A&M University (protocol#: 2005-0413). We obtained written informed consent from those participants who were ≥18 years of age at the time of enrollment or their guardians. Gentra Puregene Blood Kit (Qiagen) was used to extract genomic DNA from 3 ml whole blood following the manufacturer’s protocol. A modified protocol was used for DNA extraction from dry blood spots. Briefly, 15 ~ 20 punches of 3 mm disks were digested overnight at 55 °C by 3 ul proteinase K solution (25 mg/ml) in a 1.5 ml Eppendorf tube which containing 600 ul cell lysis solution. After digestion, samples were cool on ice for 10 min and 200 ul protein precipitation solution was added and mixed by votexing for 20 s. Mixed samples were incubated on ice for 30 min and then centrifuged for 30 min at 14,000 rpm, 4 °C. Supernatant was transfer in to a new tube containing 600 ul isopropanol and 1 ul 20 mg/ml glycogen and mixed by invert 10 times. After 48 h incubation at −20 °C, samples were centrifuged at 4 °C, 14,000 rpm for 30 min, DNA pellet was washed once in 600 ul 70 % ethanol and resuspended in 50 ul DNA hydration solution (10 mM Tris buffer). DNA was quantified using a Qubit® dsDNA BR Assay Kit (LifeTechnologies). The concentration for the tested samples were as following: A:55 ng/ul; B:49 ng/ul; C:47 ng/ul; 509:70 ng/ul; 527:61 ng/ul. 10 ng genomic DNA was then amplified using the GenomiPhi DNA Amplification kit (GE Healthcare) following the manufacturer’s standard protocol. Briefly, 1 ul of genomic DNA (diluted to 10 ng/ul) was added to 9 ul sample buffer and was heated to 95 °C for 3 min to denature the template DNA. The sample was cooled and mixed with 9 ul reaction buffer and 1 ul Phi29 enzyme and incubated at 30 °C for 16–18 h. After amplification, the Phi29 DNA polymerase was heat-inactivated by a 10-min incubation at 65 °C. The whole genome amplified (WGA) product was then quantified using the Qubit® dsDNA BR Assay Kit (LifeTechnologies), the concentration of the WGA products were as following: AW:300 ng/ul, BW:235 ng/ul, CW:330 ng/ul.

Briefly, 1.5 ug of genomic DNA was sheared to an average fragment size of 200 bp using the Covaris E220. Fragments were purified using AMPureXP beads (Beckman Coulter Inc, Brae, CA, USA) to remove small products (<100 bp), yielding 1 ug of material which was end-polished, A-tailed and adapter-ligated according to the manufacturer’s protocol. The libraries were subjected to minimal PCR cycling and quantified using the Agilent High Sensitivity DNA assay. Libraries were combined into pools for solution phase hybridization using the Illumina TruSeq™ (Illumina Inc) Exome Enrichment Kit. Captured libraries were assessed for both quality and yield using the Agilent High Sensitivity DNA assay and KAPA Library Quantification Kit. Massively parallel sequencing was performed with six samples per flow cell lane using the Illumina HiSeq2000 platform and SBS chemistry to generate 100 bp paired-end reads (2x100PE). The sequencing resulted in about 20–30X coverage across the exome. Sequences were aligned using BWA [5] to the human genome (B37). Variants were predicted using GATK [6] (V2.4) using default parameters and filtered using the GATK hard filter.

### RUFUS sequence filtering

RUFUS (http://bioinformatics.bc.edu/marthlab) is a reference independent method for identifying variants between next generation sequence data sets. It is based on a kmer-based approach that identifies sequence reads that contain unique DNA between two or more sequence libraries. The elimination of reference mapping or whole genome assembly from variant detection may reduce the rate of false positives caused by incorrect mapping without a reducing sensitivity. First, Jellyfish [7] produces kmer counts for each samples set of FASTQ files independently. RUFUS uses these counts to determine which kmers are unique to a sample. Filtered FASTQ files are generated with reads with only unique KMERs and thus reads containing a mutation compared to the comparison sequence library. Filtered FASTQ files where then used for alignment and variant calling, using the same method as the unfiltered FASTQ files.

## Results and discussion

### Technical replication

**Fig. 1:**
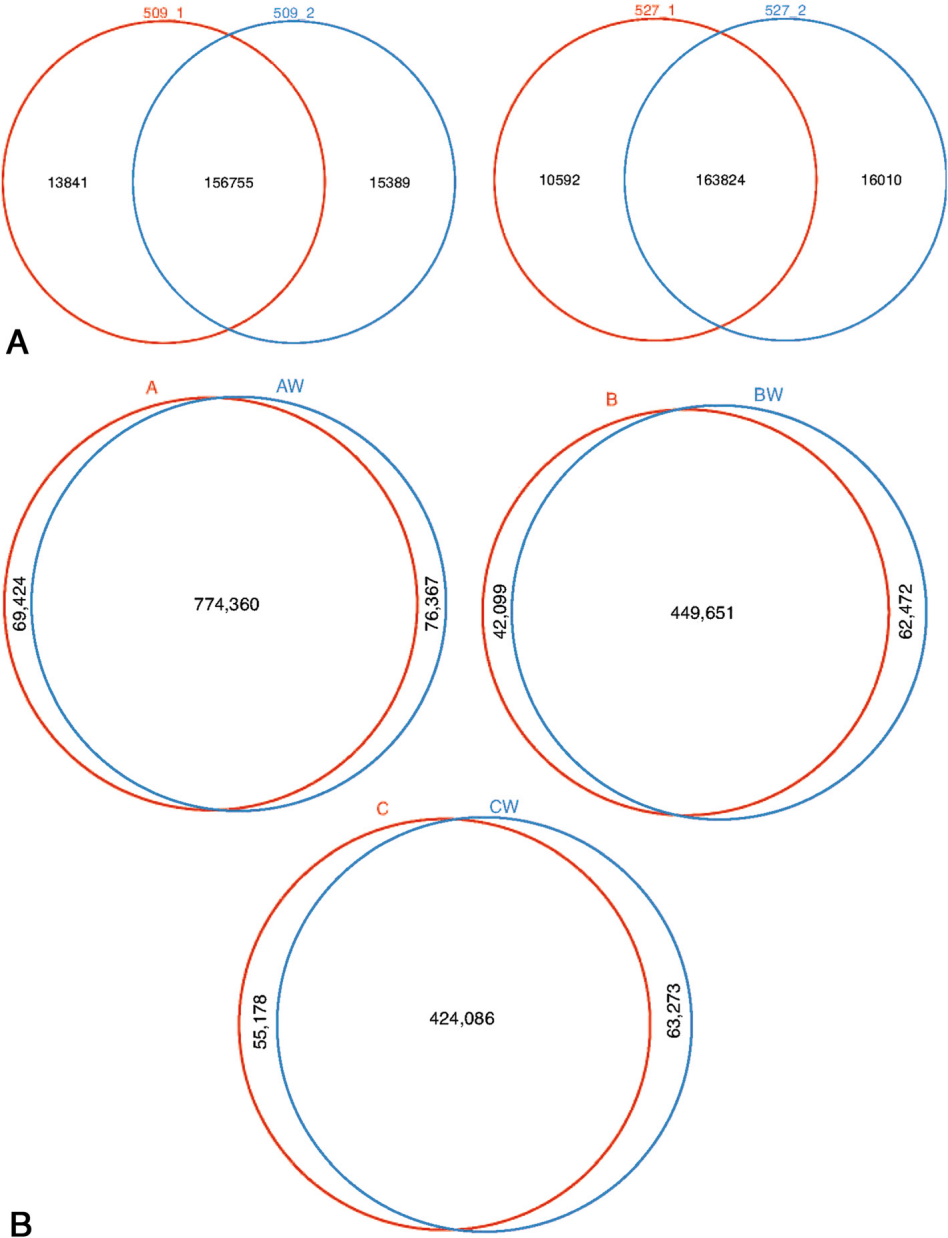
Comparison of SNVs in biological replicates. **a** Comparison of technical replicates, where both sets of sequences data from each pair is process for sequencing using the same method. **b** Comparison of biological replicates, the set labeled with the “W”, was subjected to whole genome amplification prior to library construction and sequencing

In order to determine the effect of whole genome amplification (wgaDNA) on SNV detection, we first needed to determine the concordance of SNVs in technical replicates (gDNA); in this case exome sequences from the same sample, using the same exome capture kits and processed with the same analytical pipeline. Using the classical method for SNVs determination with alignment by BWA and variants detection and filtering (hard filter) by GATK, we identified 185,985 SNVs in the two runs from sample 509, and 190,426 SNVs in the two runs from sample 527 (Fig. 1a). Consistent with previous studies [8], roughly 85 % of the SNVs are concordant between the technical replicates, with greater than 10,000 SNVs in each replicate not identified in the other. When we apply strict filtering, comparing genotypes predicted from greater than 10X depth in both samples, the rate of concordance increases to 98 %, with on average about 11 K SNVs per sample. Yet this filter removes 41 % of positions examined.

We decided to apply a reference-free sequence comparison tool, RUFUS. Using this method, we observe a dramatic improvement in technical replicate concordance. RUFUS selects sequence reads that contain differential K-mers present in one replicate but missing from the other. Variants were then called using only these reads. This removes all reads that do not contain unique sequence between the samples, thus removing reads that do not represent variation between the sample but may contribute false positives due to mapping errors. This method identifies only two discordant SNVs from the technical replicates of each sample, suggesting that much of the discordant variant calling using BWA/GATK may be introduced by mapping errors, particularly in regions of low complexity [9].

### Comparing variant calls from amplified NBS and native genomic DNA

In order to determine the effect of genome amplification, we generated sequencing libraries from the same subject where one of the replicates was amplified by whole genome amplification (wga) using Phi29 DNA polymerase. For gDNA and using “map first” method for SNV determination - comparing sequence reads to human reference genome build 37 (consisting of BWA for alignment and GATK (with a hard filter) for variant calling), we identified 920,151 SNVs in Subject A, 554,222 SNVs in Subject B and 542,537 SNVs in Subject C (Fig. 1b). Using wgaDNA, BWA/GATK SNV calls resulted in 84, 81, and 78 % concordance respectively, between variants called in the gDNA and wgaDNA from the same subject. There are 69,424 (Subject A), 42,099 (Subject B) and 55,178 (Subject C) SNVs not detected in wgaDNA, and an additional 76,367, 62,472 and 63,273 SNVs detected in wgaDNA, but not in gDNA (Fig. 1b). Using a mapping first approach, these results suggest the discordant rate of wgaDNA compared to gDNA is similar to technical replicates, (8.3, 11.2, 9.9 %) compared to on average 8.0 % in technical replicates.

To explore the filtering parameters that would decrease the number of false positives, we examined the concordant rate at 1X, 10X, 20X and 30X (Additional file 1: Figure S1), where a genotype is predicted with the given depth in both samples. Without a hard filter, the concordance rate increases from 79 to 93 %, and has 89 % of SNVs concordant for 10X, which would predict on average 15 K additional SNVs in the wgaDNA compared to gDNA. When a hard filter is imposed, the concordant rate is 85, 91, 91 and 81 % for 1X, 10X, 20X and 30X respectively. However the total number of positions meeting these depth requirements is greatly diminished, with only 17 % retained in the 10X group and less than 1 % retained in the 30X group. Of the predicted SNVs, the vast majority (96 %) are not novel in the 137 build of dbSNP. Of the SNVs detected in wgaDNA and not detected in gDNA, about 1/3 are novel for those with depths > 10X.

Using RUFUS, we identified 47, 38 and 38 K discordant SNVs when comparing the RUFUS variant calls from the wgaDNA and gDNA libraries for Subjects A, B and C, respectively. This result emphasizes that about half of discordance in calls results from the errors in alignment.

Contrasting Kmer variant call discrepancy between wgaDNA and gDNA exomes of the same subject to Kmer calls between technical replicates, we observe greater discordance between wgaDNA and gDNA than seen in technical replicate exomes (38,000–47,000 vs. 2). Nevertheless, the discordance between wgaDNA and gDNA when using a method where reads are filtered using K-mer profiles is dramatically lower than the discordance of SNVs from technical replicates when using a traditional SNV detection method, where all reads are considered (38,000–47,000 vs. 186,000–190,000). Even when using the RUFUS to filter reads which reduces overall SNV discordance in samples from the same subject, biases remain, indicating that the amplification does introduce spurious variation. Artifactual variation produced by wga represents less than 7 % of the total variation in these samples; however, the impact of this false-positive call rate on the utility of the data may be substantial.

### Capture kit variant comparison

**Fig. 2:**
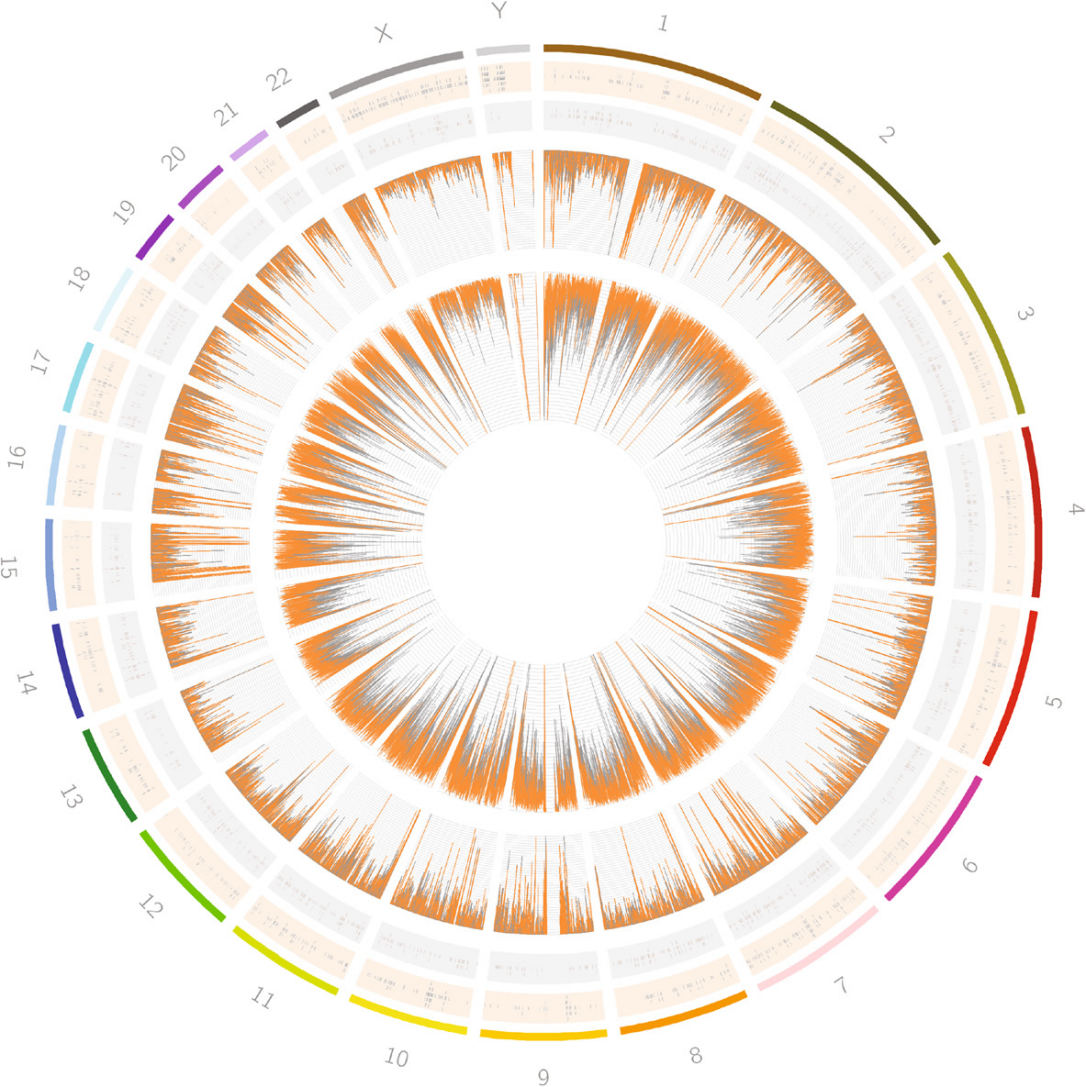
Comparison of sequence coverage and depth in biological replicates. Circos generated plot, where the outer most ring represents the chromosome of the human genome, followed by the regions of the genome with unique coverage in the Illumina capture kit (*orange*) and Agilent capture kit (*grey*), followed by a histogram of sequencing depth of each kit and a histogram of SNV density

Additionally, we wanted to know the implications of using different exome capture kits to create libraries. Therefore, we generated libraries from four subjects using the Agilent SureSelect and Illumina exome capture kit in order to determine whether capture kits produce the same variant profile. Of the SNVs predicted to be present in any of the subjects, about 31 % of the total SNVs predicted (1,012,377) were identified in samples produced by both exome capture kits. Using the GATK hard filter, only 28 % of the 353,076 filtered SNVs are concordant between capture kits. High levels of non-reproducible variant calls may therefore be obtained when attempting to compare results from exome sequencing of the same subjects when alternative capture probes/targets and their respective reagents are employed. Similar to previous findings [10], we found over 1500 regions of the genome, covered by only one of the two capture kits (Fig. 2; grey and orange rings). In overlapping regions, there are still some differences in sequencing depth (center plot), leading to differences in SNV density (Fig. 2; internal plot). These results suggest that because there are few regions that are well covered by both kits, sample preparation procedures could be more important than informatics methods in generating a list of complete SNVs. When comparing exomes in populations, researchers must be mindful of these technical challenges. Its recommended to (i) only compare SNVs detected in each dataset (i.e. samples generated using the same methods), (ii) to match sample preparation for controls and cases in disease association studies and (iii) validate with targeted resequencing.

## Conclusion

In conclusion, despite these technical challenges, we do believe that NBS are valuable resources for population genetics. We are amidst an era of sequencing and sample preparation innovation and are encouraged that newer preparation kits promise to do more with less material, which would make wga unnecessary. In the case of wga, we believe that these errors are randomly distributed in the exome (i.e. not shared between samples), therefore each variant will be rare to a whole population. In large case/control studies, the very rare variants found in one sample are often filtered.

## Additional file

Additional file 1: Figure S1. Concordance Rates. Plot represents the concordance rates between technical replicates and biological replicates (wgaDNA vs gDNA), where positions are only considered, when sequencing depth is greater than the Coverage (X-axis). Plus sign and down triangle are additionally filtered by the GATK hard filter.

## Abbreviations

NBS: Newborn bloodspot samples
DNA: Deoxyribonucleic acid
GATK: Genome Analysis Toolkit
NGS: Next-generation sequencing
SNVs: Single nucleotide variants
1000G: 1000 Genomes Project
ESP: Exome Sequencing Project
