# Supplementary figures and images for: Analysis of archived residual newborn screening blood spots after whole genome amplification
Source: BMC Genomics. 2015 Aug 13;16(1):602. doi: 10.1186/s12864-015-1747-2 (PMC4535253; doi:10.1186/s12864-015-1747-2)

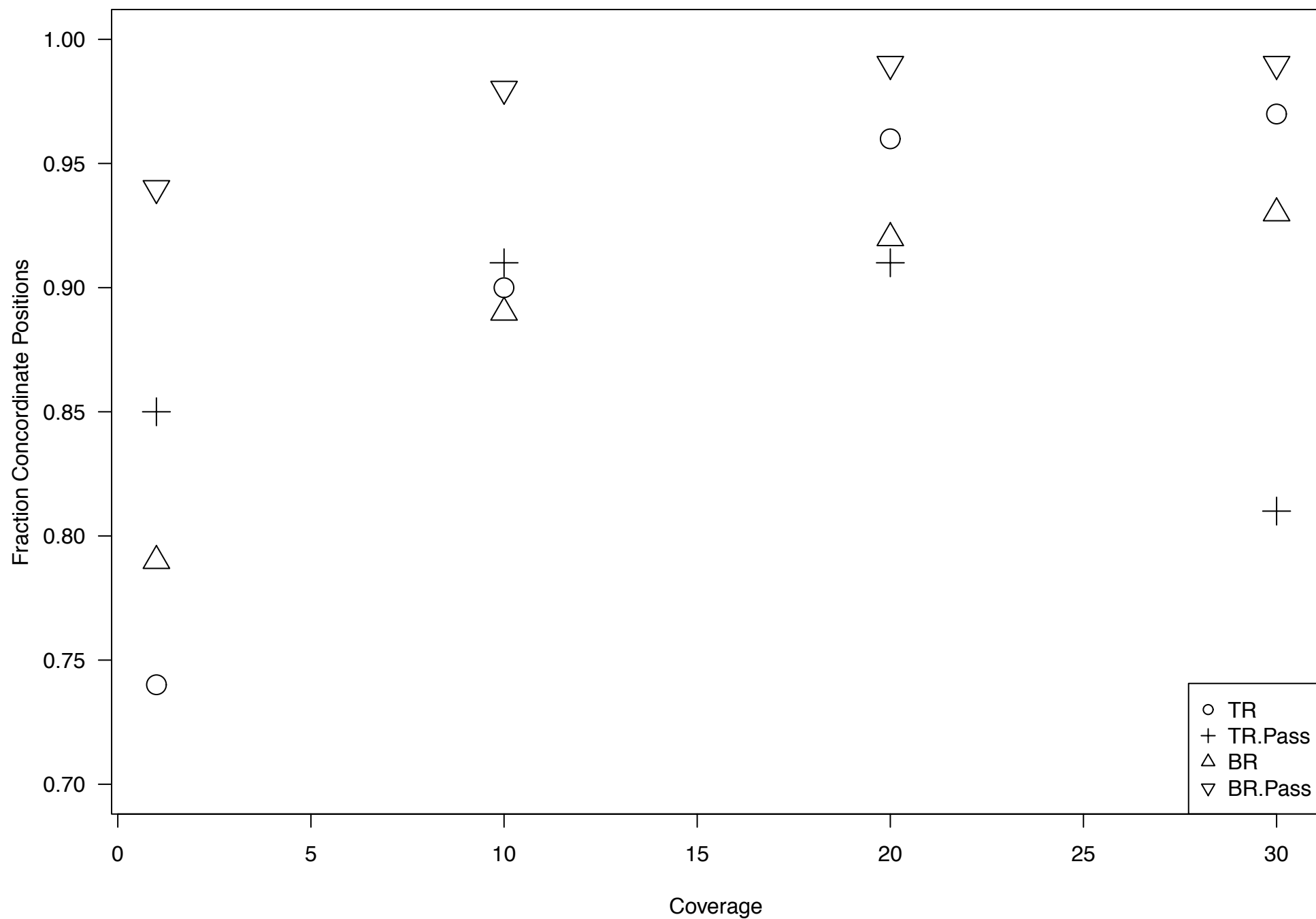

Figure S1

Supplement: Additional file 1: Figure S1. — Concordance Rates. Plot represents the concordance rates between technical replicates and biological replicates (wgaDNA vs gDNA), where positions are only considered, when sequencing depth is greater than the Coverage (X-axis). Plus sign and down triangle are additionally filtered by the GATK hard filter. [file 12864_2015_1747_MOESM1_ESM.pdf]
